# Adaptive survival mechanism to glucose restrictions

**DOI:** 10.18632/oncoscience.332

**Published:** 2016-12-16

**Authors:** Nabil Djouder

**Affiliations:** Cancer Cell Biology Programme, Growth Factors, Nutrients and Cancer Group, Spanish National Cancer Research Centre, CNIO, Madrid, Spain

**Keywords:** glucose, O-GlcNAcylation, OGT, URI, PP1Ⓒ, c-MYC, survival, diabetes, cancer, HCC

Glucose is partly metabolized through the glucose sensing hexosamine biosynthetic pathway (HBP) leading to the formation of an end product called acetylated amino sugar nucleotide uridine 5′-diphospho-*N*-acetylglucosamine (UDP-GlcNAc). UDP-GlcNAC serves as a donor substrate during *O*-GlcNAcylation (O-linked β-N-acetylglucosamine or O-GlcNAc) [1]. Serine or threonine residues of nuclear and cytoplasmic proteins are directly *O*-GlcNAcylated, competing with phosphorylation. O-GlcNAcylation is catalyzed by one unique enzyme called *O*-linked N-acetylglucosamine (O-GlcNAc) transferase (OGT). O-GlcNAcylation is cleaved and removed by another one enzyme called N-acetyl-β-D-glucosaminidase (OGA) [1]. The existence of single and unique enzymes (OGT and OGA) acting on various different substrates suggest that enzyme activity can be modulated by binding partners in response to glucose levels [1].

O-GlcNAcylation levels are very dynamic and cycles rapidly, fluctuating in response to glucose concentrations influencing cell signaling pathways [1]. O-GlcNAcylation is thus relevant to various chronic human diseases such as diabetes, cardiovascular and neurodegenerative disorders and cancer. For example, OGT promotes aneuploidy, regulates cell-cycling via HCF-1 cleavage, and participates in regulatory links between metabolic changes and carcinogenesis [2]. Changes in OGA or OGT activity and hence, in *O*-GlcNAcylation levels may occur in human breast cancer and hepatocellular carcinoma (HCC) tissues [1]. The oncoprotein c-MYC is also O-GlcNAcylated. c-MYC protein is very unstable; its levels and activity are regulated by ubiquitination and proteasomal degradation, initiated by its phosphorylation at Thr-58 by GSK3β. Thr-58 is an OGT target which regulates c-MYC stability. *O*-GlcNAcylation at Thr-58 stabilizes c-MYC, promoting tumorigenesis [1].

Unconventional prefoldin RPB5 interactor (URI) binds and modulates OGT activity in response to glucose concentrations. In presence of glucose, URI, OGT and protein phosphatase 1 gamma (PP1γ) form a heterotrimeric complex. Glucose deprivation induces anaplerotic reactions, increasing ATP/cAMP levels, thereby activating PKA which in turn, phosphorylates URI at Ser-371. Phosphorylated URI frees PP1γ from the heterotrimeric complex and, URI becomes a potent inhibitor of OGT [1]. PKA reportedly forms a mitochondrial complex with PP1 catalytic units and the pro-apoptotic Bcl-2-associated death promoter (BAD) that influences glucose homeostasis [3]. Thus, URI/OGT/PP1γ complex may integrate glucose metabolism, possibly through a mitochondrial supra-molecular complex including PKA and BAD [3,4]. Abnormal glucose metabolism and BAD requirement in glucose deprivation-induced death is reported in Bad knockout and non-phosphorylatable BAD(3SA) knockin mice [3,5]. BAD is thus an apoptotic sentinel that monitors glucose signaling. Notably, OGT overexpression in a transgenic mouse model yields a type 2 diabetes (T2D) phenotype with insulin resistance and hyperleptinemia [6]. Additionally, OGT recruitment to phosphatidylinositol 3,4,5-trisphosphate in the plasma membrane attenuates insulin signal transduction, causing insulin resistance and dyslipidaemia [7]. Thus, URI may also play a major role in glucose metabolism by regulating OGT activity.

URI-regulated OGT is reported to confer c-MYC-dependent survival functions in response to glucose fluctuations [1]. In the presence of glucose, PP1γ-bound URI increases OGT promoting c-MYC stabilization and thus, tumor progression. However, in the absence of glucose, URI inhibits OGT reducing c-MYC O-GlcNAcylation and stability allowing cells to survive. Likewise, pharmacological inhibition of OGT or its depletion reduces c-MYC levels maintaining cell survival. Thus, URI/OGT complexes are key components of an adaptive mechanism that allows tight control of c-MYC levels in cancer cells. In this regard, expression of wild-type URI in hepatocytes induces liver tumor development. According to c-MYC oncogenic functions in hepatocarcinogenesis, mice expressing non-phosphorylatable URI (S371A) in hepatocytes show higher OGT activity and c-MYC stabilization, accelerating HCC development [1].

These findings delineate an adaptive mechanism for cells to cope with metabolic stress in order to have an opportunity to survive. Prolonged exposure of cells to inadequate and low glucose concentrations induces cell survival functions allowing cells to cope with glucose restrictions or to fix a perturbed cellular homeostasis. Apoptosis of pancreatic β cells is critical in the development of T2D. Low glucose levels in β cells may thus favor OGT inhibition by URI protecting cells from death maintaining a certain cellular homeostasis. Reducing OGT activity and O-GlcNAcylation levels may be beneficial for diabetic patients, in agreement with the fact that OGT overexpression in mice causes insulin resistance and T2D [6]. URI loss-of-function in β cells may increase OGT activity, insulin resistance and development of T2D. Further research is required to better understand the role of URI in β cell functions.

OGT inhibition by URI is also an adaptive molecular mechanism enabling cancer cell survival and tumor development during glucose restrictions. Nutrients such as oxygen and glucose are delivered to various tissues via an efficient vasculature network. However, during tumor development with increasing tumor burden, an early avascular stage or collapsed vasculature inside the tumor often affect the delivery and diffusion of nutrients leading to a state of nutrient restrictions. Poor nutrient supply is demonstrated by several mathematical models to impact tumor growth, confirming experimental studies in which hypoxia supports tumor aggressiveness [1]. Not only nutrient deficiencies may play a critical role in tumor progression but may affect the metastatic process by inducing an immune adaptive response conferring an immune privilege to the tumor [8]. Moreover, anti-angiogenic agents are often used to reduce blood vessel formation and hence, the vasculature delivery capabilities, depriving nutrients to cancer cells, inducing cell death and eliminating tumor growth. Further elucidation of this mechanism in vivo will shed light on the adverse outcome of tumor development during nutrient-deprived conditions or will help to better understand why anti-angiogenic drug responses are very modest.
